# Cut them some slack: Examining the effects of slack resources on the hospital workforce

**DOI:** 10.1097/HMR.0000000000000504

**Published:** 2026-08-24

**Authors:** Frank van de Baan, Niels Hameleers, Rachel Gifford, Bram Fleuren, Dirk Ruwaard, Daan Westra

**Affiliations:** Frank van de Baan, PhD, Department of Health Services Research, Care and Public Health Research Institute (CAPHRI), Faculty of Health, Medicine and Life Sciences, Maastricht University, Maastricht, The Netherlands. E-mail: f.vandebaan@maastrichtuniversity.nl. Niels Hameleers, MSc, Department of Health Services Research, Care and Public Health Research Institute (CAPHRI), Faculty of Health, Medicine and Life Sciences, Maastricht University, Maastricht, The Netherlands. Rachel Gifford, PhD, Department of Health Services Research, Care and Public Health Research Institute (CAPHRI), Faculty of Health, Medicine and Life Sciences, Maastricht University, Maastricht, The Netherlands. Dirk Ruwaard, PhD, Md, Department of Health Services Research, Care and Public Health Research Institute (CAPHRI), Faculty of Health, Medicine and Life Sciences, Maastricht University, Maastricht, The Netherlands. Daan Westra, PhD, Department of Health Services Research, Care and Public Health Research Institute (CAPHRI), Faculty of Health, Medicine and Life Sciences, Maastricht University, Maastricht, The Netherlands; Bram Fleuren, PhD, Department of Work and Social Psychology, Faculty of Psychology and Neuroscience, Maastricht University, Maastricht, The Netherlands

**Keywords:** buffers, capacity building, efficiency, health workforce, pandemic preparedness, resource allocation, resource constraints, workforce crisis

## Abstract

**Background:**

Resource and capacity constraints can hamper the functioning of health care organizations and adversely affect the workforce, particularly during a shock. To mitigate the workforce crisis and enhance the ability to deal with shocks, slack resources (surplus resources accumulated by organizations) may be beneficial.

**Purpose:**

This paper seeks to identify how slack may benefit hospital staff in terms of reduced absenteeism and turnover, and how slack may buffer against the negative effects of shocks on staff.

**Methodology/Approach:**

Using a nationwide dataset including annual reports of all Dutch hospitals from 2017 to 2021, this paper studies the relationships between three types of slack resources and absenteeism and turnover rates of hospitals. Specifically, linear mixed-effects modeling is used to estimate direct effects of slack resources and to test how COVID-19 (vs. non-COVID-19 years) moderates these resources’ effects based on 342 hospital-year combinations.

**Results:**

The analyses reveal a significant curvilinear U-shaped relationship between absorbed slack (i.e., staff levels) and turnover rates. In addition, unabsorbed slack (i.e., financial reserves) and potential slack (i.e., capacity for additional loans) buffered significantly against the increasing effect of COVID-19 on absenteeism rates.

**Practice Implications:**

Our study shows that incentivizing health care organizations to acquire slack resources can enable them to reduce turnover rates and, during shocks, mitigate shocks’ adverse impact on absenteeism rates. As such, this studu suggests a need to reconsider the common policies and organizational practices of constraining resources to reduce costs.

Health care organizations are often confronted with resource and capacity constraints, which can have multifaceted drawbacks (Diefenbach, 2009). Studies show that such constraints can hamper organizations’ abilities to innovate (Diefenbach, 2009) or adapt to (rapid) change (Mazzucato & Kattel, 2020), and limit the quality and accessibility of services (Page et al., 2024). Moreover, resource and capacity constraints can burden health care workers since staff need to do more with fewer resources (OECD, 2023), which can lead to absenteeism and turnover of staff (Cavalcante de Oliveira et al., 2023). Shocks (i.e., high-consequence events that have a major disruptive effect) can worsen these issues, as staff struggle to meet work demands that exceed the status quo (Mithani, 2020). For example, the heavy burden of the COVID-19 pandemic on health care organizations exacerbated the high absenteeism and turnover in the sector (Azzopardi-Muscat et al., 2023). The challenges emerging from resource and capacity constraints lead scholars and policymakers to call for transformations to ensure the functioning of health care systems and organizations going forward (OECD, 2023). The introduction of slack resources (i.e., surplus resources accumulated by organizations) has been suggested, but the extent to which slack resources can enhance the functioning of health care organizations remains unclear (Ellis et al., 2023).

The value and efficacy of slack resources continue to be subject to scholarly debate (Bentley & Kehoe, 2020; Vanacker et al., 2017). Some studies posit a positive relationship between slack resources and organizational outcomes, including financial performance (Carnes et al., 2019), innovation (Chen & Miller, 2007; Greve, 2003), and the ability to engage in strategic change (Bentley & Kehoe, 2020). Other studies find such relationships to be curvilinear (Tan & Peng, 2003) or negative depending on the type of slack (Vanacker et al., 2017). While these findings shed light on the slack and organizational performance relationship, our understanding of the value of slack remains limited (Bentley & Kehoe, 2020; Mount, 2024). Studies often describe slack vaguely, without clarifying the specific type or characteristic of the slack resource being studied (Mount, 2024). When studies do specify slack, they often only include unabsorbed slack (Mount, 2024), which is problematic since the implications for organizational outcomes can vary depending on the specific type of slack assessed (Vanacker et al., 2017). In addition, studies typically examine the influence of slack resources during nonshock periods (Essuman et al., 2022). During a shock, slack is believed to serve a distinct role by buffering against disruptions (Bourgeois, 1981), yet this role warrants further study due to the limited number of empirical studies conducted (Essuman et al., 2022). Lastly, studies tend to focus on financial performance or innovation as measures of organizational performance (Bentley & Kehoe, 2020; Vanacker et al., 2017). While these are considered key performance indicators, organizational performance encompasses a variety of factors that can determine organizations’ health and success, including quality of products and services (Bentley & Kehoe, 2020) and the scope and capabilities of human resources (Hancock et al., 2017). Exploring the relationship between slack resources and human resource outcomes can be particularly useful in light of workforce shortages present in the health care sector (Azzopardi-Muscat et al., 2023).

To advance the knowledge on the value of slack resources for organizations, this paper studies the effects of slack resources on the hospital workforce before and during a shock (i.e., the COVID-19 pandemic). We conceptualize COVID-19 as a large-scale, exogenous shock that provides an empirical context to examine how slack resources buffer organizations against external disruptions. Drawing on literature on organizational slack resources, we use a longitudinal multilevel approach to examine the relationship between three types of slack resources (i.e., unabsorbed, absorbed and potential slack) and absenteeism and turnover rates of hospitals in the Netherlands between 2017 and 2021. Unabsorbed slack refers to reserves readily available for use, such as savings; absorbed slack refers to administrative resources, such as the amount of staff; and potential slack refers to the capacity to obtain additional resources, such as borrowing capacity (Depino-Besada et al., 2025). The study is guided by two main research questions: *What is the relationship between slack resources and absenteeism and turnover rates of hospitals?* And: *To what extent do slack resources buffer against the effects of shocks on absenteeism and turnover rates of hospitals?*

## Theory

### Organizational Slack Resources

In their seminal book, Cyert and March (1963) define slack resources as the “*disparity between the resources available to the organization and the payments required to maintain the coalition*” (Cyert & March, 1963; 36). This definition is among the first in scholarly discussions on slack and positions slack resources as the resources—often considered in terms of time, budget or capacity—that exceed the minimum that is necessary for completing a specific task, activity, or project. Bourgeois (1981) expanded the definition of slack by combining various definitions of slack proposed since the work of Cyert and March (1963). He defines slack as “*that cushion of actual or potential resources which allows an organization to adapt successfully to internal pressures for adjustment or to external pressures for change in policy, as well as to initiate changes in strategy with respect to the external environment*” (Bourgeois, 1981; 30). Thus, slack resources allow organizations to redeploy buffers toward adapting strategies or pursuing new strategic directions, and they can act as a buffer during disruptions, allowing organizations to use them to meet the new demands caused by these disruptions.

There are three dominant perspectives concerning how slack resources influence organizational outcomes (Mohr & Young, 2012). First, strategic management literature, specifically studies using the Resource-Based View of the firm (Helfat & Peteraf, 2003), considers slack to be beneficial to organizations as it allows them to pursue new strategies that increase performance and enables them to cope with sudden internal and external shocks (Tan & Peng, 2003). Second, and contrastingly, neoclassical economics views slack resources as waste that should be minimized to achieve maximum efficiency (Sharfman et al., 1988). Similarly, from an agency perspective, slack is considered negative since management could use these resources for personal (e.g., in the pursuit of power or job security) rather than organizational gain (Depino-Besada et al., 2025). Third, uniting both perspectives, the intermediary view perceives slack to be beneficial for performance to a certain extent (Sharfman et al., 1988). Here, scholars propose an inverted U-shaped curvilinear relationship, where some slack can enable the pursuit of new strategies and allow for flexibility, but excessive slack would negatively impact outcomes as it leads to inefficiencies and complacency (Depino-Besada et al., 2025).

The literature identifies three main types of slack resources, namely unabsorbed, absorbed and potential slack (Bourgeois, 1981), with unabsorbed slack being the most studied type (Mount et al., 2024). Unabsorbed slack consists of resources that are readily available within an organization and not yet used, such as cash flows, reserve funds, and retained earnings (Bourgeois & Singh, 1983). It is typically operationalized through liquidity ratios, comparing assets and inventories to liabilities (Carnes et al., 2019). Absorbed slack refers to the administrative resources existing within an organization—such as the amount of staff, time available to staff, and amount of training—beyond what is needed for operational demands (Greve, 2003). Contrary to unabsorbed slack, absorbed slack resources are not readily available to the organization as they are incorporated in operations, yet they can be recovered (through negotiation and reconfiguration) (Greve, 2003). Absorbed slack is often operationalized as the number or costs of employees to firm sales or assets (Carnes et al., 2019). Lastly, potential slack resources are resources organizations can acquire through debt financing (Geiger et al., 2019). Potential slack is seen as external slack as it does not reside within the organization, but instead can be acquired through loans when deemed necessary (Bourgeois & Singh, 1983). As such, it captures organizations’ existing financial leverage and future borrowing capacity (Carnes et al., 2019). Potential slack is typically operationalized through debt-to-equity or debt-to-assets ratios (Carnes et al., 2019).

### The Relationship Between Slack Resources and Absenteeism and Turnover Rates

Although the relationship between slack resources and absenteeism and turnover has not been empirically assessed, there has been some theorizing of a link based on the notion that slack could allow organizations to deploy resources toward strategies to reduce stress, improve working conditions and sustain staff and enhance their well-being (Ellis et al., 2023; Zinn & Flood, 2009). For example, Zinn and Flood (2009) posit in their commentary that health care organizations might mitigate issues pertaining to turnover and absenteeism by deploying resources toward investments in facilities, less stressful work conditions or expanding benefits. Furthermore, Ellis et al. (2023) propose that absorbed slack resources enable health care organizations to deploy additional staff to departments where they are needed most. As such, absorbed slack resources could serve as a protective mechanism for staff well-being as it reduces the workload for staff (Ellis et al., 2023).

Overall, in light of the literature, we predict that slack resources can influence absenteeism and turnover rates of organizations. Considering the divergent perspectives among slack scholars regarding the relationship between slack resources and organizational outcomes, with some proposing a curvilinear relationship, we propose the following:

Hypothesis 1a. In the absence of shocks, organizations with greater amounts of slack resources (i.e., absorbed, unabsorbed and potential slack) will have lower absenteeism and turnover rates compared with organizations with less amounts of slack resources.

Hypothesis 1b: The effect of slack resources on absenteeism and turnover rates is curvilinear, following a U-shaped pattern.

### Shocks and the Buffering Capabilities of Slack Resources

Shocks are characterized as “*high-consequence events that have a major disruptive effect*” (OECD, 2023, p. 21). Regarding the recent COVID-19 pandemic, scholars posit that more than during previous shocks, the pandemic can be seen as a shock that particularly had an impact on the humans *within* the organization (Collings et al., 2021; Newman et al., 2023). As such, it has also been described as a life-threatening event, as the first priority following the shock was the physical and emotional well-being of staff (Mithani, 2020). Fearing for their own health, uncertainty about the severity and longevity of the crisis, changes in how work could be done, and—particularly in the health care sector—high demand for services put a significant strain on employees (Collings et al., 2021). Being at the forefront of the crisis, studies show that health care workers experienced increased workload (Fronteira et al., 2024), a lack of rest (Lotta et al., 2021), and inadequate working conditions (Fronteira et al., 2024). Such factors have in turn been found to be associated with withdrawal behavior, including absenteeism (Fronteira et al., 2024) and intention to leave the profession (Lotta et al., 2021; Fronteira et al., 2024). In light of the effects that shocks can have on the workforce, we predict the following:

Hypothesis 2a: Shocks have a positive effect on absenteeism and turnover rates, such that during a shock, organizations have higher absenteeism and turnover compared with before the shock.

Given the severity and frequency of shocks, there has been increasing attention toward the need for organizations to be better able to absorb shocks (Barasa et al., 2018; Mithani, 2020). Slack resources could prove valuable to organizations considering its shock-absorbing capabilities (Mithani, 2020). Specifically, scholars argue that slack allows organizations to deploy buffers toward strategies to mitigate the disruptive effects of shocks (Depino-Besada et al., 2025). For example, Leuridan and Demil (2022) show how slack was produced and used at a critical care unit during various stages of unanticipated events. The authors detail how particularly absorbed slack allowed the unit to deal with unexpected workflows, whereby often specialists from various departments of the hospital were called in to assist when extra staff was needed during various situations, including emergencies and a crisis (Leuridan & Demil, 2022). Contrary to a nonshock period, scholars deem a curvilinear relationship between slack and organizational performance unlikely as the impact of shocks tend to be greater than what the excess resources can compensate for (Depino-Besada et al., 2025).

Similar to the nonshock period, research so far has not examined the influence of slack on the workforce during a shock. Considering its characteristics, slack resources could be valuable during shocks as the buffers allow organizations to engage in practices aimed at lowering the disruptive effects of shocks on the workforce. For example, following Leuridan and Demil (2022), having absorbed slack (i.e., excess staff) could allow hospitals to redeploy staff from departments where the workload is lower to departments where there is a high workload, thereby distributing the burden more evenly. In addition, organizations could use unabsorbed or potential slack (i.e., financial resources) to hire more staff as a means to further lower the workload. These financial buffers could then also be used as financial remuneration to compensate for the inadequate working conditions caused by the shock. Such practices have been shown to be beneficial for sustaining staff during shocks (Cavalcante de Oliveira et al., 2023).

Taking into account the shock-absorbing capabilities of slack resources, we propose the following:

Hypothesis 2b: Organizational slack resources buffer the positive effect of shocks on absenteeism and turnover rates, such that organizations with more slack resources have lower increases in absenteeism and turnover during a shock than those with fewer slack resources.

## Methods

This is a longitudinal study, spanning a 5-year period between 2017 and 2021.

We use a national dataset containing annual data of all hospitals in the Netherlands. We focus on hospitals since these entities were heavily affected by the pandemic (Gifford et al., 2022a). In accordance with the Dutch Medical Research Involving Human Subjects Act (Wet medisch-wetenschappelijk onderzoek met mensen; WMO), this study—based solely on publicly available data and involving no direct interaction with or intervention in human participants—does not fall under the scope of the WMO and is therefore exempt from review by a Dutch Medical Research Ethics Committee (METC).

### Setting

This study was conducted in the Netherlands, where general, academic, and specialized hospitals are the main organizations providing curative care, alongside independent treatment centers (providing outpatient services only) (Kroneman et al., 2016). All hospitals are private, nonprofit entities (i.e., foundations) that are selectively contracted by private health insurers on a yearly basis (Kroneman et al., 2016). Care provided to patients is reimbursed by these insurers on a DRG-like basis (Kroneman et al., 2016). The hospital workforce consists of 14.9 staff per 1000 inhabitants, just above the OECD average (14.7) (OECD, 2021). On January 1, 2022, there were 72 active hospitals in the Netherlands, of which eight were academic hospitals (CIBG, n.d.). Academic hospitals provide more specialized care services than general hospitals (Kroneman et al., 2016). In addition, they have a major role in research and education (Kroneman et al., 2016). Overall, the average operating margin for hospitals was 1.7% in the Netherlands in 2021 (EY, 2022).

This study used the COVID-19 pandemic as an example of an exogenous shock to test the buffering effect of slack resources on workforce outcomes. During the first wave of the pandemic (i.e., March to May 2020), hospitals almost entirely scaled down regular care to have sufficient capacity for COVID-19 patients (Gifford et al., 2022a). For the remainder of the crisis, hospitals constantly balanced COVID-19 capacity with regular care capacity to ensure that care needs could be met as much as possible (Gifford et al., 2022a). When needed, COVID-19 patients were also transferred to other hospitals with available capacity (Gifford et al., 2022a).

### Data and Sample

We used a national dataset of annual report data of Dutch health care organizations. The dataset is publicly available (CIBG, n.d.). Among others, it includes financial data (e.g., revenue and liabilities), capacity data (e.g., number of available beds and total amount of patient days), and workforce data (e.g., absenteeism and turnover). Overall, our sample includes general and academic hospitals, totaling 342 hospital-year observations. We excluded specialized hospitals and independent treatment centers since these vary greatly between countries in structure and function, lowering comparability and generalizability of findings (Lega & De Pietro, 2005). In addition, hospitals that were not operational during the entire study period (e.g., due to mergers or bankruptcy) or had missing data for 2 or more years were excluded. Hospitals with missing data for only 1 year were retained in the sample, as linear mixed-effects models can still be applied in such cases.

### Variables

In our analysis, we included absenteeism rates and turnover rates as outcome variables. Absenteeism rates per organizations could directly be retrieved from the annual report datasets. In line with the literature, absenteeism rates were operationalized as the number of absent days within the organization divided by the total number of available workdays in a given period (in this case, a year) (Davey et al., 2009). Following operationalization in previous literature (Heavey et al., 2013), turnover rates were calculated as a ratio of the total number of FTE (full-time equivalent) staff leaving the organization in a given year, divided by the total number of FTE staff the organization had that year. Both the numerator and denominator could be derived from the annual report datasets. Using rates as dependent variables ensured that outcomes were adjusted for differences in the size of the organization (i.e., larger organizations likely having a higher total absolute number of absent days and a higher absolute number of FTE staff leaving the organization).

Our main independent variables of interest were unabsorbed, absorbed and potential slack. These variables were operationalized following the work of Geiger et al. (2019), a recent study assessing the relationship between slack resources and hospital outcomes. Unabsorbed slack was operationalized using the quick ratio, calculated as current assets minus inventories divided by current liabilities, where ratios below one are typically an indicator of poor financial health of an organization (Geiger et al., 2019). Absorbed slack was operationalized using man-hours (in FTE) per adjusted patient day. In line with Geiger et al. (2019), adjusted patient day was calculated as the amount of clinical patient days divided by the ratio of patient revenues to total revenues. Here, higher rates indicate more FTE per patient day, whereas lower rates indicate less FTE per patient day. Lastly, potential slack was operationalized using the debt-to-sales ratio. This was calculated as the total debts divided by all revenue from care delivery. Since debt is the numerator, as opposed to the other slack variables, higher rates (above 1) indicate lower rates of potential slack as the ability to acquire more financial resources decreases. To be able to analyze a potential curvilinear effect of slack resources, we furthermore created quadratic slack variables by multiplying each slack variable by itself. Next to that, we included the COVID-19 pandemic as a year dummy (coded as 1 in 2020 and 2021, and as 0 in 2017–2019) to analyze the effect of this shock on the relationship between slack and workforce outcomes.

In line with literature on the relationship between slack resources and hospital performance, hospital size (measured in terms of total amount of FTE working at the organization at the end of each year) and status as an academic hospital were included as control variables to adjust for confounding (Geiger et al., 2019). Furthermore, the year (ranging from 2017 to 2021) was included to account for the longitudinal character of the data. While studies primarily conducted in the United States typically also include rural/urban location as a control variable, we did not do so since the Netherlands is densely populated, with a relatively equal spread of citizens across the country (Irwin et al., 2009).

### Data Analysis

All data were analyzed using IBM-SPSS. Descriptive statistics were calculated to explore frequencies, means and standard deviations. The associations between slack and the outcome measures were estimated using linear mixed-effects models to account for repeated measures within organizations. To best account for the dependence of the repeated measures within organizations, the first-order autoregressive covariance structure (AR1) was used to model the data. This structure is in line with the longitudinal nature of the data, as it assumes that observations closer in time (e.g., in 2017 and 2018) are correlated more strongly than those further apart in time (e.g., in 2017 and 2021). We tested the fit of multiple other covariance structures using likelihood ratio tests, and the AR1 structure fitted the data best. For both dependent variables (i.e., absenteeism rates and turnover rates), we ran three models; (i) a base model testing the effect of unabsorbed slack, absorbed slack, potential slack, and COVID-19 year on the outcomes, controlling for year, hospital size and academic status, (ii) a model testing the curvilinear effect of slack by adding the quadratic slack variables to the base model, and (iii) a model testing the effect of slack during periods of exogenous shock, by adding the interaction between the three slack types and the COVID-19 year dummy to the base model. A *p* values under .05 were considered statistically significant.

To ensure the validity and consistency of findings, robustness checks and sensitivity analyses were performed. These analyses included running separate isolated models in which the effects of single slack variables and single interaction terms on absenteeism and turnover rates were assessed. This way, we could rule out that the significant effects identified were only present due to model complexity instead of the single effect. These checks yielded similar results.

## Results

**FIGURE 1:**
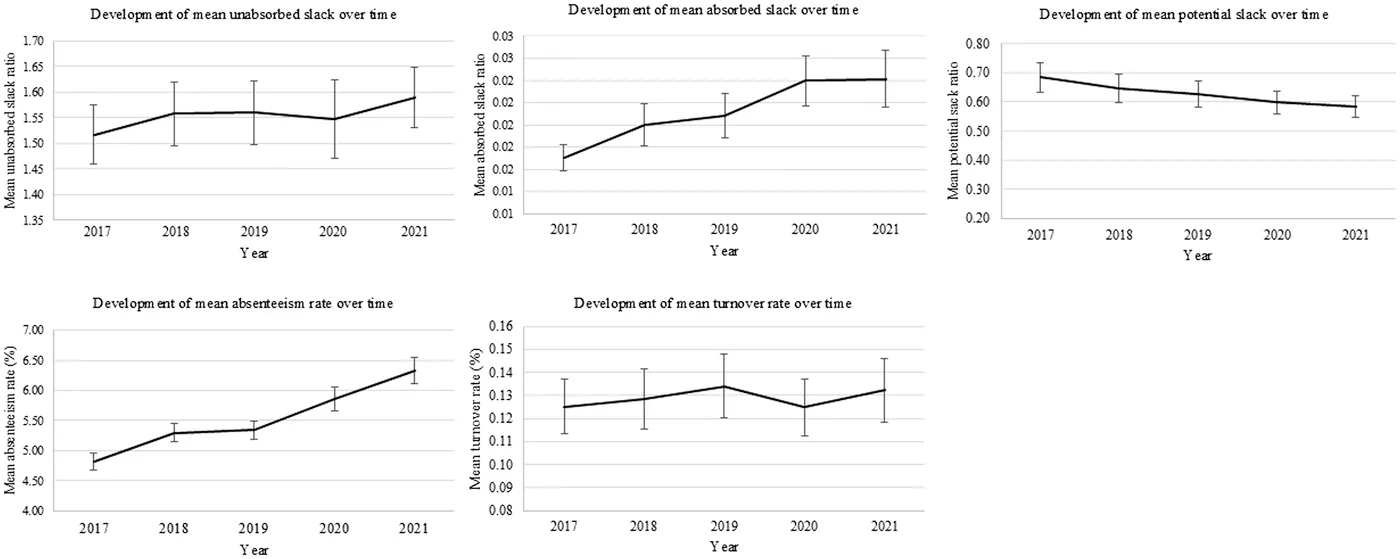
Means and standard deviations of slack variables, absenteeism, and turnover rates are plotted over time.

Table 1 presents the descriptive statistics of our sample. As the table indicates, the average amount of staff per hospital (in FTE) increased by over 10% from 2017 to 2020 (i.e., from 2,732 FTE to 3,083 FTE), slightly decreasing in 2021 (3,020 FTE). Hospitals varied greatly in size. In 2021, for example, the smallest hospital had a staff size of 270 FTE, while the largest hospital consisted of 10,408 FTE that year. Regarding the slack variables, Table 1 and Figure 1 show that, on average, all three slack types increased during the study period. Unabsorbed slack (i.e., current assets minus inventories divided by current liabilities) increased from a 1.52 ratio in 2017 to a 1.62 ratio by 2021. Absorbed slack (i.e., amount of FTE adjusted per patient days) increased from a 0.016 ratio in 2017 to a 0.020 ratio by 2019 and then stabilized to 0.023 in 2020 and 2021. The relatively sharp increase of absorbed slack during the COVID-19 years (i.e., 2020 and 2021) seemed to be the result of a decrease in the number of patient days during those years. The potential slack ratio (i.e., debt to sales) decreased from 0.68 in 2017 to 0.58 by 2021, indicating an increased potential to acquire more financial resources (i.e., increased potential slack level). Similar to the slack levels, the average absenteeism rates also increased during the study period. Turnover rates decreased during the first year of the pandemic, only to return to 2019 levels by the second year of the pandemic.

**TABLE 1:** Descriptive statistics

	2017		2018		2019		2020		2021	
Variables	*N*	Mean (SD) | Min–Max | Q1–Q2–Q3	*N*	Mean (SD) | Min–Max | Q1–Q2–Q3	*N*	Mean (SD) | Min–Max | Q1–Q2–Q3	*N*	Mean (SD) | Min–Max | Q1–Q2–Q3	*N*	Mean (SD) | Min–Max | Q1–Q2–Q3
Hospitals^a^	68	0.12 (0.32)	70	0.11 (0.32)	69	0.12 (0.32)	66	0.12 (0.33)	69	0.12 (0.32)
Total amount of staff (FTE)	68	2,732 (2,285)	70	2,894 (2,277)	69	2,962 (2,252)	66	3,083 (2,243)	69	3,020 (2,288)
COVID year	68	0.00 (0.00)	70	0.00 (0.00)	69	0.00 (0.00)	66	1.00 (0.00)	69	1.00 (0.00)
Unabsorbed slack	68	1.52 (0.25) | 0.97–2.25 | 1.34–1.46–1.69	70	1.58 (0.27) | 0.98–2.45) | 1.37–1.51–1.74	69	1.58 (0.26) | 0.99–2.42 | 1.38–1.51–1.72	66	1.60 (0.25) | 1.04–2.18 | 1.40–1.54–1.67	69	1.62 (0.25) | 1.05-2.22 | 1.42-1.57-1.73
Absorbed slack	68	0.016 (0.01) | 0.01–0.03 | 0.01–0.02–0.02	70	0.019 (0.01) | 0.00–0.05 | 0.01–0.02–0.02	69	0.020 (0.01) | 0.01–0.05 | 0.01–0.02–0.02	66	0.023 (0.01) | 0.01–0.05 | 0.01–0.02–0.03	69	0.023 (0.01) | 0.01-0.08 | 0.02-0.02-0.03
Potential slack	68	0.68 (0.21) | 0.32–1.31 | 0.52–0.67–0.78	70	0.65 (0.21) | 0.33–1.25) | 0.49–0.64–0.75	69	0.63 (0.19) | 0.31–1.12 | 0.50–0.61–0.73	66	0.60 (0.16) | 0.33–1.08 | 0.48–0.57–0.66	69	0.58 (0.16) | 0.32–0.99 | 0.47–0.56–0.70
Absenteeism rate (%)	67	4.82 (0.57) | 3.30–6.51 | 4.50–4.72–5.19	70	5.30 (0.66) | 3.94–6.89) | 4.85–5.29–5.64	69	5.34 (0.66) | 3.85-7.80 | 4.97-5.26-5.66	66	5.84 (0.77) | 4.28–8.40 | 5.25–5.70–6.49	69	6.33 (0.91) | 4.20–9.40 | 5.69–6.21–6.89
Turnover rate (%)	65	12.52 (4.75) | 3.83–25.56 | 9.52–11.52–14.59	69	13.03 (5.38) | 3.20-34.99 | 10.09–12.16–14.59	67	13.40 (5.71) | 1.32–33.06 | 9.56–11.93–14.75	65	12.74 (4.95) | 1.97–27.23 | 9.45–11.91–14.47	67	13.82 (5.17) | 3.05–34.38 | 10.61–12.56–15.69

^a^ *Note*. The mean rate presents the percentage of academic hospitals compared with the total number of hospitals included. For each year, eight academic hospitals were included.

Tables 2 and 3 present the results of the multilevel models. Model 1 was used to test the relationship between slack resources and absenteeism and turnover rates for a nonshock period. Contrary to hypothesis 1a, absenteeism and turnover rates were not significantly correlated with the level of unabsorbed, absorbed or potential slack of hospitals, as shown by model 1 in Tables 2 and 3. However, as shown in Table 3, model 2 revealed a significant curvilinear relation between absorbed slack and turnover rates (*β*=5380.07, *p*<.01, 95% CI=1527.58–9232.56). Thus, hypothesis 1b was partially confirmed.

**TABLE 2:** Results of linear mixed-effects models predicting the effect of slack resources on absenteeism rates

Parameters	Null Model (*N*=342)	Model 1 (*N*=342)	Model 2 (*N*=342)	Model 3 (*N*=342)
Intercept		6.28 (5.30–7.26)***	6.78 (5.67–7.89)***	5.93 (4.88–6.98)***
Control variables				
Academic hospital		−0.82 (−1.44, −0.20)**	−0.76 (−1.39, −0.13)***	−0.73 (−1.36, −0.11)*
Size (FTE)		0.00 (0.00–0.00)	0.00 (0.00–0.00)	0.00 (0.00–0.00)
Independent variables				
Year		0.24 (0.15–0.33)***	0.23 (0.14–0.32)***	0.24 (0.15–0.33)***
COVID-19		0.23 (0.03–0.42)*	0.22 (0.03–0.42)*	1.45 (0.52–2.38)**
Year × COVID-19		0.29 (0.03–0.24)*	0.24 (0.05–0.42)*	0.21 (0.29–0.39)*
Unabsorbed slack		−0.39 (−1.01, 0.22)	−0.77 (−1.52, 0.03)*	−0.17 (−0.83, 0.48)
Absorbed slack		9.99 (0.00–19.98)	16.35 (−1.07, 33.77)	8.08 (−5.19, 21.34)
Potential slack		−0.11 (−0.98, 0.77)	−0.67 (−1.76, 0.42)	0.16 (−0.73, 1.05)
Unabsorbed slack quadratic			0.57 (−0.56, 1.70)	
Absorbed slack quadratic			−246.29 (−721.10, 228.52)	
Potential slack quadratic			1.68 (−0.35, 3.71)	
Two-way interaction				
Unabsorbed slack × COVID-19				−0.80 (−1.39, −0.22)**
Absorbed slack × COVID-19				1.65 (−12.08, 15.39)
Potential slack × COVID-19				−1.35 (−2.22, −0.48)**
ICC	.29	.58	.60	.59
AIC	849.30	606.91	586.09	590.87
BIC	856.95	618.33	597.48	602.27

*Note.* Coefficients are reported with 95% confidence intervals in parentheses. Model 1: no interaction; model 2: no interaction, quadratic effect; nodel 3, interaction.

N refers to the amount of hospital-year observation included in each model.

* *p*<.05.

** *p*<.01.

*** *p*<.001.

**TABLE 3:** Results of linear mixed-effects models predicting the effect of slack resources on turnover rates

Parameters	Null Model (*N*=333)	Model 1 (*N*=333)	Model 2 (*N*=333)	Model 3 (*N*=333)
Intercept		18.25 (10.70–25.80)***	18.90 (10.60–27.20)***	19.15 (10.97–27.28)***
Control variables				
Academic hospital		0.23 (−4.49, 4.94)	0.35 (−4.34, 5.03	0.46 (−4.31, 5.23)
Size (FTE)		0.00 (−0.00, 0.00)	0.00 (0.00–0.00)	0.00 (0.00–0.00)
Independent variables				
Year		0.59 (−0.16, 1.33)	0.78 (0.02–1.53)*	0.68 (−0.08, 1.44)
COVID-19		−1.28 (−3.03, 0.48)	−1.13 (−2.86, 0.60)	−2.29 (−9.79, 5.21)
Year × COVID-19		0.35 (−1.21, 1.90)	0.00 (−1.56, 1.57)	0.23 (−1.35, 1.80)
Unabsorbed slack		−2.79 (−7.49, 1.90)	−4.19 (9.73–1.34)	−3.38 (−8.47, 1.70)
Absorbed slack		−3.65 (−83.14, 75.84)	−164.14 (−300.97, −27.31)*	−49.18 (−157.42, 59.05)
Potential slack		1.04 (−5.60, 7.68)	−3.13 (−11.15, 4.90)	0.78 (−5.99, 7.56)
Unabsorbed slack quadratic			−0.64 (−9.23, 7.95)	
Absorbed slack quadratic			5380.07 (1527.58–9232.56)**	
Potential slack quadratic			12.67 (−2.70, 28.05)	
*Two-way interaction*				
Unabsorbed slack x COVID-19				0.50 (−4.19, 5.20)
Absorbed slack x COVID-19				68.50 (−46.22, 183.22)
Potential slack x COVID-19				−1.60 (−8.54, 5.34)
ICC	.40	.46	.47	.46
AIC	1979.99	1965.26	1928.33	1945.88
BIC	1987.60	1976.61	1939.65	1957.19

*Note.* Coefficients are reported with 95% confidence intervals in parentheses. Model 1: no interaction; model 2: no interaction, quadratic effect; model 3, interaction.

*N* refers to the amount of hospital-year observation included in each model.

* *p*<.05.

** *p*<.01.

*** *p*<.001.

We further tested the influence of a shock on absenteeism and turnover rates. As shown in Table 2, hypothesis 2a was partially confirmed as Model 3 indicated that absenteeism rates were significantly higher during the COVID-19 years (*β*=1.45, *p*<.01, 95% CI=.52–2.38). However, turnover rates during the COVID-19 years did not differ significantly from the levels before the crisis, as indicated by the insignificant COVID-19 variable in model 3 of Table 3. The potential buffering relationship between slack resources and absenteeism and turnover rates was also examined. We found partial confirmation for hypothesis 2b, as model 3 indicated a significant effect of unabsorbed slack resources on absenteeism rates during a shock (Table 2). Specifically, the model showed a significant interaction effect between the COVID-19 parameter and unabsorbed slack on absenteeism rates (*β*=−.80, *p*<.01, 95% CI=−1.39, −.22). Furthermore, model 3 showed there also was a significant interaction between the COVID-19 parameter and potential slack on absenteeism rates (*β*=−1.35, *p*<.01, 95% CI=−2.22, −.48). Specifically, hospitals with higher potential slack had relatively lower absenteeism rates than those with lower potential slack during COVID-19 while the opposite was true before COVID-19.

## Discussion

In this paper, we applied linear mixed-effects models to examine the relationship between slack resources and absenteeism and turnover of the hospital workforce during periods with and without shock. The results show that absorbed slack resources in the form of staff levels exhibit a curvilinear relationship with turnover rates of hospitals. Specifically, moderate rates of absorbed slack are associated with lower turnover, whereas lower and higher rates of absorbed slack are associated with higher turnover. Our results further show that unabsorbed slack (in the form of financial reserves) and potential slack (the ability to take on loans) are associated with lower absenteeism rates of hospitals during a shock. Overall, our study underscores the beneficial attributes of slack resources for the workforce of organizations, depending on the type and quantity of slack resources, as well as the condition of the external environment. In the following paragraphs, we reflect on our findings.

This study identifies absorbed slack as a factor that can significantly influence turnover rates of hospitals. The identified curvilinear relationship partly confirms our first hypothesis and provides further support for the notion that slack operates in an optimum with organizational outcomes (Sharfman et al., 1988). A potential rationale for the observed curvilinear relationship could be that maintaining broader staff levels enables organizations to mitigate workload (Ellis et al., 2023) and enhance overall appeal of the organization (Heavey et al., 2013), yet an excessive surplus may diminish organizational attractiveness as there could be lower cohesion (Hancock et al., 2017) or insufficient allocation of meaningful work (Humphrey et al., 2007). Contrary to our hypothesis, it is noteworthy that we did not find unabsorbed slack and potential slack to affect turnover rates. It might be the case that hospitals were reluctant to utilize these resources toward strategies to reduce turnover since they operate in resource-constrained environments where investments in human resources are not prioritized (National Academies of Sciences Engineering and Medicine, 2019). Furthermore, regarding potential slack, Mount et al. (2024) note that organizations often have limited discretion in allocating these resources due to restrictions imposed by lenders. Consequently, even if hospitals wanted to dedicate additional resources towards strategies to sustain staff, restrictions on their use might have hindered such investments.

Our findings further indicate that unabsorbed and potential slack can serve as a buffer against a shock, limiting increases in absenteeism rates. This partially confirms our second hypothesis and advances literature on the buffering capabilities of slack resources. Our finding might be explained by the fact that unabsorbed and potential slack allow organizations to use monetary resources towards strategies to sustain staff during a shock. Research shows that during the COVID-19 pandemic, health care organizations increased their spending on strategies to do so, for example, through the creation of welfare facilities and financial remuneration for additional work (Cavalcante de Oliveira et al., 2023). Notably, we found that unabsorbed and potential slack were relevant during the pandemic, but not in a nonshock period. This might suggest that the willingness to deploy slack resources may be contingent on the severity of external pressures. While hospitals operate in a resource-constrained environment, slack does generally exist, albeit in narrow margins. Given these narrow margins, hospitals may be reluctant to utilize them during nonshock periods, when workforce issues appear more manageable, but become more willing to utilize them in response to a shock.

Our finding that absorbed slack does not mitigate increased absenteeism during a shock may stem from the limited number of purposes it can be used for and the efforts required to reconfigure it (Mount et al., 2024). While our findings contradict Leuridan and Demil (2022), who demonstrate the important role of absorbed slack within the critical care unit, they emphasize that the value of absorbed slack lies in its proper use and reconfiguration. In their case, practices can be considered quite institutionalized. Staff perceived slack practices as integral to the department’s mission, actively secured additional resources, and frequently reconfigured slack when deemed necessary (Leuridan & Demil, 2022). Contrastingly, during COVID-19, hospitals struggled to recover absorbed slack due to the sudden disruption and lack of experience doing so (Gifford et al., 2022a). In addition, the specific care needed for COVID-19 patients, coupled with workforce specialization, hampered the ability to match staff with patients (Lotta et al., 2021). This supports the notion that it is difficult to consider the value of absorbed slack without taking into account organizations’ ability to sufficiently recover and use absorbed slack.

We did not find slack resources to buffer against increased turnover rates caused by a shock. This could be attributed to the longitudinal nature of turnover, characterized by an initial period of intention to leave preceding the actual departure (Hancock et al., 2017). Consequently, the impact of the COVID-19 pandemic on turnover may not have been fully evident in our data, which extends only until 2021. Research suggests a high level of commitment among hospital staff to work during the early stages of the pandemic, whereby the commitment decreased only later on during the pandemic (Gifford et al., 2022b). This could have had a temporal effect on turnover rates, which could also explain the decreased turnover rate in our data for 2020 compared with 2019. As such, this temporal influence may have masked a potential buffering effect of slack on turnover, and make that only after an extensive period of time an effect could be observed.

### Theoretical Contributions

This study offers two important theoretical contributions. First, it advances slack literature by demonstrating that slack resources influence workforce outcomes. As such, we expand understanding of the value of slack beyond traditionally studied outcomes such as financial performance (Bentley & Kehoe, 2020), innovation (Greve, 2003), or—specific to health care—quality of care (Mohr & Young, 2012). Second, the fact that we find the same type of slack to have a different effect on organizational outcomes depending on the condition of the external environment provides an important nuance to the debate on the value of slack. Our findings corroborate recent calls made by Mount et al. (2024) to not regard slack in a vague general sense, but to be critical of the differences in the type of slack and outcome studied, whereby we add that the condition of the external environment is an important factor to consider when determining the potential value of slack.

## Implications

At the health system level, our findings highlight the value of incentivizing health care organizations to establish reserves as a means to address workforce challenges. Although hospitals operate in financially constrained environments, they do tend to retain some reserves. For example, the average operating margin for Dutch hospitals was 1.7% in 2021. However, given the narrowness of these margins, organizations may be reluctant to deploy these reserves. Policy measures at the system level that enable hospitals to maintain broader margins may therefore strengthen their capacity to respond effectively to workforce challenges and shocks. This also aligns with calls to move away from prevailing policies that constrain resources in the pursuit of cost reduction (Diefenbach, 2009; Mazzucato & Kattel, 2020). Building on prior research that shows how these constraints can have negative effects (e.g., Mazzucato & Kattel, 2020; Page et al., 2024), our study shows how broader margins can have a positive impact, particularly for the workforce. The importance of establishing buffers becomes further pronounced in light of the dynamic environment health care organizations must operate in, characterized by uncertainty and the emergence of shocks (OECD, 2023). Alongside persistent challenges, such as aging and increased multimorbidity among the population, health care organizations increasingly face new shocks, including wars, climate change and infectious disease outbreaks (OECD, 2023). As such, they must enhance their ability to adapt to changes and absorb shocks (OECD, 2023). A sufficient workforce forms a crucial component for this, and our findings indicate that slack resources can contribute to mitigating the negative effects of a shock and protect the workforce.

At the organizational level, our findings suggest that hospitals should build buffers as a means for sustaining a sufficient workforce. A potential paradox emerges in the case of absorbed slack; absorbed slack can increase the workforce of an organization (through lower turnover of staff), but for organizations to create absorbed slack, a larger workforce seems to be needed (to create buffers among staff). Recruiting additional staff is challenging given current workforce shortages (Azzopardi-Muscat et al., 2023). Therefore, organizations should focus on creating more latitude among their current employees. This could be achieved by decreasing the administrative burden and the number of tasks assigned to staff. Although this has been deemed difficult to achieve due to the heavily institutionalized nature of work, the COVID-19 pandemic has shown that tasks can be lifted without compromising care quality, safety or cost-effectiveness (Sinsky & Linzer, 2020).

### Limitations

This study is subject to several limitations. First, our findings are derived from analyses of Dutch hospital data, whereby the limited number of hospitals in the Netherlands limits the statistical power and generalizability of our study. Second, this study makes use of ratios, which may complicate the interpretation of results (Certo et al., 2020). Nonetheless, our ratios were developed in line with slack literature (e.g., Bentley & Kehoe, 2020; Geiger et al., 2019), and their use is common within the health care management field in general. Third, our study design does not allow for causal claims to be made. As such, while this study shows that there is a relationship between slack and workforce outcomes, future research is needed to further understand this relationship. Scholarship could particularly benefit from mixed-methods studies to examine the effect of slack on workforce outcomes, and the underlying mechanisms that are at play in order for slack to have these effects. Fourth, the observed absenteeism and turnover rates could be influenced by other factors than slack. These include organizational climate, work pressure, task allocation and compensation (Davey et al., 2009; Heavey et al., 2013). COVID-19-specific factors could have also played a role. For example, research has shown that absenteeism rates were also driven by infection among staff (Maltezou et al., 2023). Our data did not allow us to include such variables, and we were therefore unable to control for them in this study. Fifth, absorbed slack is inherently difficult to operationalize in health care settings, as higher staffing intensity may reflect either excess capacity or legitimate clinical needs. Although our measure of man-hours per adjusted patient-day aligns with prior research, it may not fully distinguish productive utilization from idle capacity. Similarly, while our proxy for potential slack follows established approaches, coverage-based indicators such as the interest coverage ratio or debt service coverage ratio may better capture external resource potential and creditworthiness. Future studies employing alternative proxies for absorbed and potential slack are needed to further clarify the relationship between slack and absenteeism and turnover rates.
